# Solvent Welding-Based Methods Gently and Effectively Enhance the Conductivity of a Silver Nanowire Network

**DOI:** 10.3390/nano13212865

**Published:** 2023-10-29

**Authors:** Zhaoxi Zhu, Xiaolu Wang, Dan Li, Haiyang Yu, Xuefei Li, Fu Guo

**Affiliations:** 1 Faculty of Materials and Manufacturing, Beijing University of Technology, Beijing 100124, China; zhuzx@emails.bjut.edu.cn (Z.Z.); lidan@bjut.edu.cn (D.L.); yuhaiyang@emails.bjut.edu.cn (H.Y.); lixuefei2022@emails.bjut.edu.cn (X.L.); 2 Key Laboratory of Advanced Functional Materials, Ministry of Education, Beijing 100124, China; 3 School of Mechanical Electrical Engineering, Beijing Information Science and Technology University, Beijing 100192, China

**Keywords:** silver nanowire, transparent conductor, nanowelding, plasma treatment, Joule heating

## Abstract

To enhance the conductivity of a silver nanowire (Ag NW) network, a facile solvent welding method was developed. Soaking a Ag NW network in ethylene glycol (EG) or alcohol for less than 15 min decreased the resistance about 70%. Further combined solvent processing via a plasmonic welding approach decreased the resistance about 85%. This was achieved by simply exposing the EG-soaked Ag NW network to a low-power blue light (60 mW/cm^2^). Research results suggest that poly(vinylpyrrolidone) (PVP) dissolution by solvent brings nanowires into closer contact, and this reduced gap distance between nanowires enhances the plasmonic welding effect, hence further decreasing resistance. Aside from this dual combination of methods, a triple combination with Joule heating welding induced by applying a current to the Ag NW network decreased the resistance about 96%. Although conductivity was significantly enhanced, our results showed that the melting at Ag NW junctions was relatively negligible, which indicates that the enhancement in conductivity could be attributed to the removal of PVP layers. Moreover, the approaches were quite gentle so any potential damage to Ag NWs or polymer substrates by overheating (e.g., excessive Joule heating) was avoided entirely, making the approaches suitable for application in devices using heat-sensitive materials.

## 1. Introduction

Silver nanowire (Ag NW) networks exhibit exceptional flexibility, electrical conductivity, optical transmittance, and the advantage of facile synthesis and deposition, positioning them as a competitive candidate to replace indium tin oxide in various applications [1,2,3,4,5]. The polyol method has mainly been applied to the synthesis of Ag NWs for its relatively gentle reaction conditions and facile processing [6,7,8]. Although synthesized Ag NWs can be directly deposited onto substrates to form conductive films, the resistance of a Ag NW film is mainly dependent on the resistance at the Ag NW junctions, which is much greater than the resistance of the Ag NW itself, hindering their applications. The contact resistance mainly originates from residual insulating surfactant on the nanowire surface, such as poly(vinylpyrrolidone) (PVP), an indispensable capping agent in polyol methods that hinders the metal–metal contact at Ag NW junctions [9,10,11]. In order to improve the conductivity of a Ag NW network, various post-treatment processes have been developed to remove residual PVP to ensure metal–metal contact and increase the metallic contact area.

Thermal welding methods, which have been widely investigated and applied, elevate the temperature to higher than 150 °C to simultaneously evaporate the residual PVP and weld junctions of adjacent Ag NWs. However, such high temperatures limit the use of heat-sensitive polymer substrates that are necessary for flexible electronics. Moreover, a Ag NW network quickly fails upon thermal treatment as a result of thermally induced spheroidization of Ag NWs [9,12,13]. In order to remove the residual PVP and weld junctions of Ag NWs without thermal damage, methods that avoid overheating during thermal welding processes have been proposed. For example, intense pulsed ion beam irradiation (IPIB) [14] generates enormous heat in small areas to weld the Ag NW network, but the short-lasting time of 200 ns helps the bulk of the substrate remain at a low temperature. Another welding method that uses a similar principle is high-power laser welding [15]. However, these high-energy beam methods provide practical welding at the cost of expensive equipment, and the efficiency is affected as welding is limited to small areas.

Larger-area welding can be conducted by light-induced plasmonic welding, in which the heat generation comes from the excitation of localized surface plasmon resonance on Ag NW junctions. During this welding process, the optical radiation couples with plasmonic modes to generate extreme local heating at nanometer-scale small gaps between the upper and lower Ag NWs at junctions. The heat generation strongly depends on the gap distance as more heat is produced with the stronger optical field confinement that occurs when the gap becomes smaller. Light sources with intensities of 30–200 $W/{cm}^{2}$ were used for welding Ag NWs. Such self-limited plasmonic welding methods can selectively generate large amounts of heat at the Ag NW junctions without significantly affecting substrates [16,17,18,19,20,21]. However, the plasmonic welding method still needs a high-intensity light source with a specific wavelength, making this precision technology hard to use extensively.

Another selective junction heating thermal method is current-assisted localized Joule heating, an efficient approach with low power consumption that welds the Ag NWs with electromigration. When voltage is applied at both ends of the Ag NW film, the current passing through the minor contact points at the junctions with the residual insulating surface ligands generates enough power to weld all the contacts of the Ag NWs across the entire film. During this process, electromigration occurs at the high-current-density regions in the nanowire network, enhancing welding and reshaping the contact into a ripened contact geometry [22]. However, precision control of the current is needed in current-assisted localized Joule heating as the Joule heating effect may cause failure of Ag NW networks via overheating [23].

Although various thermal welding methods have been proposed as discussed above, precision control on thermal input and welding time is still needed to prevent thermal damage of the Ag NWs and substrates. To avoid potential overheating issues, room-temperature chemical welding methods, which do not require any external energy, have also been developed. Such welding strategies are usually performed via the reduction of Ag^+^ ions to form Ag atoms as solder, which can effectively solder the junctions of Ag NWs with the use of various chemical reagents such as sodium halide salt solutions [24], silver nitrate and ascorbic acid [25], ionic liquid-type reducing agents [26], and polydopamine [27]. Although room-temperature welding has been developed, these chemical welding methods face problems with the addition of complex chemicals, and residual impurities easily control the Ag NW network.

Without any chemical solutes, solvents themselves can also be used to remove residual PVP layers on Ag NWs. For instance, it was reported that by using methanol to wash Ag NWs with a vacuum filtering method, the thickness of the PVP layer could be reduced from 4 to 0.5 nm by increasing the frequency of washing from one to five times [12]. It should be pointed out that this method involves cleaning Ag NWs before depositing them into networks, and bare Ag NWs easily aggregate and cannot be stored for long, which can affect later applications. Recently, a convenient post-processing solvent welding method called capillary-force-induced welding was proposed [28,29,30]. By simply spraying moisture on Ag NW films, the Ag NW junctions are filled with water droplets; the evaporation of these droplets provides GPa level pressure between the adjacent Ag NWs to increase their contact area. Also, the residual PVP can be partially detached by the moisture, which shortens the Ag NW junction gap and benefits the cold welding process. The difficulty in such methods is the need for the sophisticated fixing of the volatilization rate and substrate hydrophobicity in order to produce optimal welding conditions.

As illustrated above, although great efforts have been made on improving welding methods and have achieved some good results, there are still issues including risk of thermal damage and needs for high power, expensive instruments, and complex control. Thus, effective but gentle Ag NW welding strategies are needed to meet requirements in practical applications.

**Figure 1:**
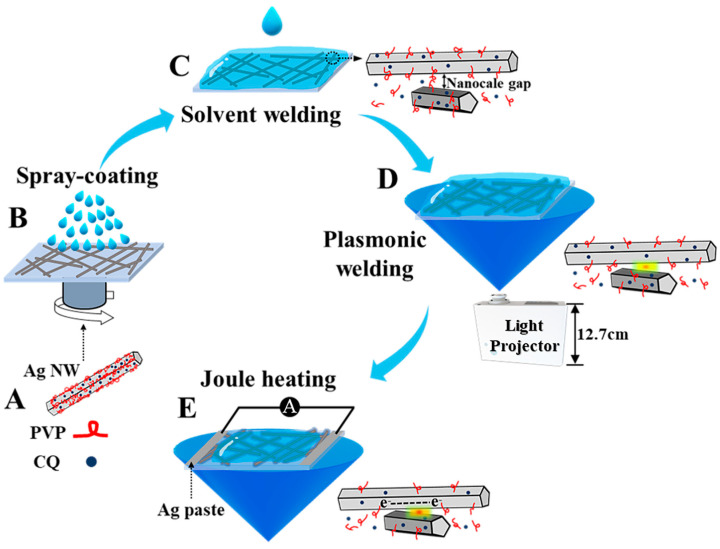
Schematic of Ag NW synthesis with the capping of PVP (red line) and CQ (blue dot) (**A**), fabrication of Ag NW networks via a spray-coating process (**B**), solvent welding process and the corresponding state of Ag NW junctions (**C**), solvent-based plasmonic welding process (**D**), and combined solvent-based plasmonic and Joule heating welding process (**E**).

In this work, we found that a simple soaking treatment that was the addition of ethylene glycol (EG) or alcohol liquid film to the Ag NW network would effectively enhance the overall Ag NW network conductivity. In our previous work, we synthesized Ag NWs with the addition of poly(vinylpyrrolidone) (PVP) using a camphorquinone (CQ)-mediated polyol method. Then, we developed a facile solvent welding method in this work, presented in Figure 1C. A combined solvent-based plasmonic welding method was later developed for further enhancing the conductivity (Figure 1D). This method was realized by projecting a low-power blue light (60 mW/cm^2^) on the Ag NW network that was immersed in EG. Finite element simulation was also performed to investigate the localized surface plasmonic resonances and heat generations of Ag NW junctions, with the focus on the simulation of junctions with the presence of EG. For further improving Ag NW network conductivity, a Joule heating strategy was utilized by simultaneously applying current to the Ag NW network (Figure 1E). Performances of these welding approaches on lowering the materials’ resistance and influences on structures of nanowire junctions were thoroughly studied. And this research provided a feasible strategy of combining different methods to meet stringent requirements in practical applications, for example, using thermal-sensitive polymers.

## 2. Materials and Methods

### 2.1. Materials

Silver nitrate (AgNO_3_, 99.85%) was purchased from Guangdong Guanghua Sci-Tech Co., Ltd. (Shantou, China). Poly(vinylpyrrolidone) (BR, K90) with an average molecular weight of 360,000 g/mol was obtained from Shanghai Yuanye Bio-Technology Co., Ltd. (Shanghai, China). Iron(III) chloride hexahydrate (97%) and camphorquinone (99%) were purchased from Alfa Aesar. Ethylene glycol (AR) and ethanol (AR) were sourced from Da Mao Chemical Reagent Factory (China). All reagents were used without further purification.

### 2.2. Synthesis of Ag NWs

A one-pot polyol method was employed to synthesize silver nanowires (Ag NWs) of different dimensions, based on the CQ-mediated polyol approach for silver nanoparticles with improvements [31]. Ag NWs of different dimensions were synthesized by varying the CQ concentrations. In a typical synthesis flow, 0.249 g of CQ and 0.120 g of PVP were dissolved in 15 mL of EG. After complete dissolution, 0.150 g of AgNO_3_ was added and dissolved in the mixture. Subsequently, 1.8 mL of iron(III) chloride hexahydrate (600 µM in EG solution) was introduced and vigorously stirred for 4 min. The solution was then heated to 150 °C and stirred at 300 rpm for 5 h. The resulting product was washed with ethanol, followed by centrifugation at 4000 rpm for multiple cycles. The product was then redispersed and stored in ethanol before use. To synthesize Ag NWs of various dimensions, CQ molar ratios relative to AgNO_3_ were adjusted within the range of 0.5–1.7.

### 2.3. Fabrication of Ag NW Networks

To fabricate the Ag NW networks using the spray-coating method, glass substrates (thickness: 0.23 mm) and polyethylene terephthalate (PET) substrates (thickness: 0.1 mm) were employed. The substrates were cleaned with ethanol and deionized water using an ultra-sonicator for 3 min, followed by rotation at 50 rpm using the KW-4A desktop homogenizer (purchased from SETCAS Electronics Co., Ltd., Hong Kong, China) to ensure uniform deposition. The synthesized Ag NWs were formulated into Ag NW ink with ethanol solvent (7.5 mg/mL) and subsequently sprayed onto the clean substrates at room temperature. The schematic of the spray-coating process is depicted in Figure 1B.

### 2.4. Welding of Ag NW Networks

In the solvent welding process of the Ag NW networks, EG and ethanol were individually used to weld the fabricated Ag NW networks for 1 to 60 min. Following the welding process, the residual EG was removed by rinsing with ethanol, and the Ag NW films were rapidly dried at room temperature in a drying box. A schematic of the solvent welding process is presented in Figure 1C.

In the solvent-based plasmonic welding process, the fabricated Ag NW networks were exposed to blue light emitted by a commercial advanced laser phosphor display laser projector (GaN based, purchased from Appotronics Inc., Milpitas, CA, USA), which emitted continuous-wave laser light. After removal of its color wheel, the laser projector emitted blue light with a central wavelength of 445 nm. Prior to welding, the Ag NW networks were pre-wetted with EG. Subsequently, the Ag NW networks were welded using blue light with an energy intensity of $60\pm 2\;mW/{cm}^{2}$ and exposure time varying from 1 to 60 min. A schematic of the solvent-based plasmonic welding process is displayed in Figure 1D.

After solvent-based plasmonic and Joule heating welding of Ag NW networks, a conductive silver ink pen (purchased from Shenzhen Jingzhe Technology Co., Ltd., Shenzhen, China) was employed to draw two 3 mm wide silver channels onto the fabricated Ag NW film. A Keithley 2100 6 1/2 Digit Multi-meter, a direct current power supply with a bias voltage of 20 V, was used to apply a controlled current to the Ag NW network through the deposited silver electrodes. Throughout the welding process, the current was constantly limited within the range of 0.05–0.20 A to run the real-time resistance measurements. The process of combined solvent-based plasmonic and Joule heating welding is illustrated in Figure 1E.

### 2.5. Characterization

Scanning electron microscopy (SEM) and energy-dispersive spectroscopy (EDS) analyses were performed using a Hitachi SU8020 scanning electron microscope. Transmission electron microscope (TEM) images, selected-area electron diffraction (SAED) patterns, and high-resolution TEM (HRTEM) images were acquired with a JEM Hitachi 2100F instrument. X-ray photoelectron spectra (XPS) measurement was taken by Thermo Scientific Escalab 250Xi. The sheet resistances of samples were measured using a four-probe method employing a ROOKO FT-340 tester. The changes in resistance were monitored using a two-probe Keithley 2100 6 1/2 Digit Multi-meter. Optical transmittance was measured using a UH-4150 UV-vis-NIR spectrophotometer from Hitachi. The laser intensity was measured using a Newport 843-R power meter. For distances and surrounding mediums, the commercial COMSOL program was employed to simulate the plasmonic effect caused by 445 nm blue light at Ag NW junctions under different gaps. The interaction between light and nanostructure was described by the classical Maxwell’s equations, and the electric field distribution was calculated from the Helmholtz wave equation [32]. The generated heat induced by light absorption was directly related to the imaginary part of the dielectric constant of material [33,34]. The details about the simulation model are included in the Supplementary Materials (Figure S6).

## 3. Results and Discussion

### 3.1. CQ-Mediated Polyol Synthesis of Ag Nanowires with Different Dimensions

Our prior research demonstrated that CQ acted as a small-molecule capping agent during the synthesis of silver nanoparticles and was particularly effective in capping nanocrystals with small sizes compared to PVP. The capping of CQ has significant effects on controlling the product morphologies as it can effectively stabilize small-sized nanocrystals [31]. In this research, we proposed a CQ-mediated polyol method for synthesizing Ag NWs with controlled dimensions and decreased the residual amount of PVP.

**Figure 2:**
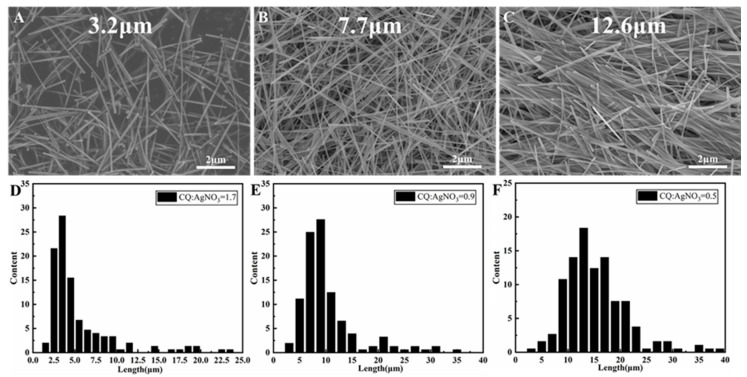
SEM images (**A**–**C**) and corresponding statistical length distributions (**D**–**F**) of Ag NWs synthesized at different CQ to AgNO_3_ molar ratios: (**A**,**D**) 1.7, (**B**,**E**) 0.9, (**C**,**F**) 0.5. All experiments were conducted under a PVP to AgNO_3_ molar ratio of 1.2:1. Average lengths are indicated in the respective images.

Figure 2 presents SEM images and corresponding statistical length distribution of Ag NWs synthesized using various CQ: AgNO_3_ molar ratios while maintaining consistent PVP and AgNO_3_ concentrations. Ag NWs with average lengths of 3.2, 7.7, and 12.6 µm were synthesized with CQ: AgNO_3_ molar ratios of 1.7, 0.9, and 0.5, respectively, as shown in Figure 2A–F. The average length of Ag NWs increased in inverse proportion to the CQ concentration. Furthermore, the primary length distributions of Ag NWs expanded as the CQ concentration decreased, with ranges of 2.5–5, 5–12, and 8–17 µm, respectively, in Figure 2A–C. These results indicated that CQ plays a role in constraining the growth of Ag NWs in the longitudinal direction and contributed to enhancing the uniformity in their size.

**Figure 3:**
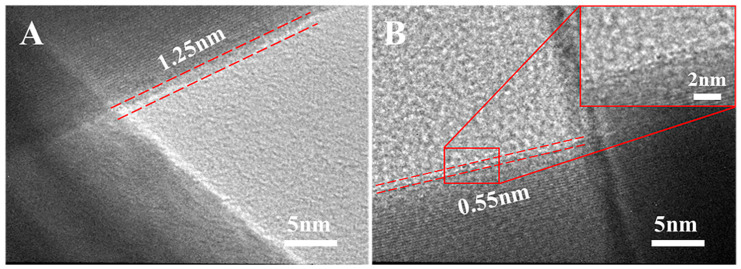
TEM images at junctions of Ag NWs synthesized without CQ (**A**) and with a CQ: AgNO_3_ molar ratio of 1.7 (**B**), showing the thickness of PVP layers. The inset is a locally magnified image.

The diameters of the synthesized Ag NWs exhibited minor changes with varying CQ: Ag NO_3_ molar ratios. As displayed in Figure S1, the average diameter of Ag NWs decreased with decreasing CQ concentration. Ag NWs with average diameters of 89, 86, and 82 nm were respectively synthesized with CQ: AgNO_3_ molar ratios of 1.7, 0.9, and 0.5. Consequently, the dimension control of Ag NWs was achieved by simply adjusting the CQ concentration. Ag NWs with lengths of 3.2, 7.7, and 12.6 µm were respectively synthesized with diameters of 89, 86, and 82 nm. Besides morphology changes, the CQ capping also led to a reduction in the thickness of PVP layers on the as-synthesized Ag NWs. Specifically, the PVP layer thickness decreased from 1.25 nm to 0.55 nm with an increase in CQ: AgNO_3_ molar ratios from 0 to 1.7, depicted in Figure 3.

We hypothesized that the decrease in Ag NW length with an increase in the amount of CQ was owing to the improved stability of small nanocrystals and the capping of CQ. This effect induced a higher number of Ag NWs with the same amount of silver source, resulting in less silver precursor available per Ag NW. A similar mechanism has been proposed previously to explain the dimension control of Ag NWs in other synthetic methods [35,36]. It is suggested that CQ capping decreases the thickness of PVP layers because CQ will take positions on the surfaces of silver nanoparticles, reducing the amount of attached PVP during synthesis.

The Ag NWs synthesized at a CQ: AgNO_3_ molar ratio of 0.5 were expected to offer advantages for fabricating Ag NW films, as these Ag NWs exhibited the most extended length of 12.6 µm among those obtained via CQ-mediated polyol synthesis and thinner PVP layers compared to Ag NWs synthesized using traditional polyol methods.

### 3.2. Solvent-Based Plasmonic Welding of Ag Nanowire Networks

**Figure 4:**
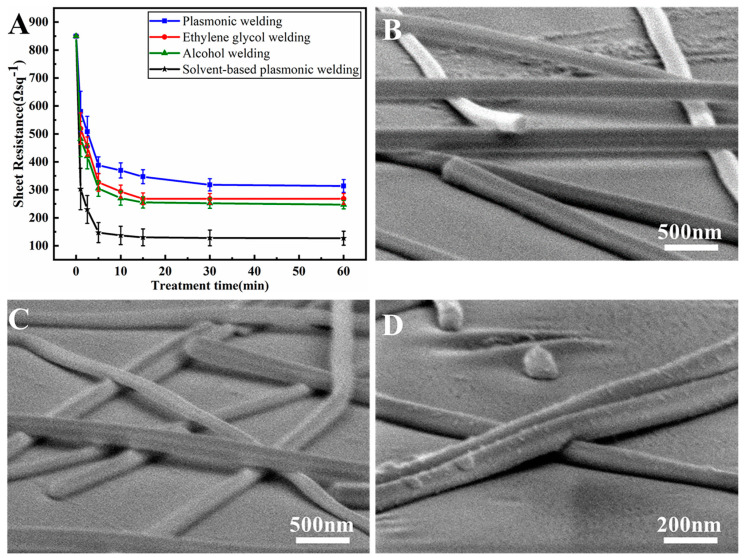
(**A**) Sheet resistance of Ag NW films at various welding times using different methods: plasmonic welding (blue), EG welding (red), alcohol welding (green), and solvent-based plasmonic welding (black). The initial sheet resistance of the Ag NW films was 850 $\Omega sq^{-1}$. SEM images of the Ag NW networks (**B**) before welding, (**C**) after welding for 30 min through the EG welding method, and (**D**) after solvent-based plasmonic welding.

In this study, Ag NW films were fabricated using a spray-coating method at room temperature. To reduce the resistance of the fabricated Ag NW films, a solvent welding process involving soaking the Ag NW in EG or ethanol for treatment of 1–60 min was developed. Both EG and ethanol were chosen owing to their ability to dissolve CQ and PVP at room temperature. As depicted in Figure 4A, the solvent welding method using EG and ethanol effectively reduced the sheet resistance of Ag NW films. The sheet resistance notably decreased within the first 15 min, with only slight changes beyond that time. The ultimate sheet resistance of EG and ethanol welded samples was 268 and 247$\;\Omega sq^{-1}$, respectively. This novel room-temperature solvent welding approach, achieved through a simple soaking process, offered a convenient way to reduce the resistance of flexible Ag NW films with polymer substrates. However, further enhancements are required to improve the efficiency of solvent welding.

Besides solvent welding, we developed a room-temperature plasmonic welding method employing low-energy blue light. Figure 4A illustrates the reduction in resistance during the plasmonic welding process. The energy of the blue light source, operating at $60\pm 2\;mW/{cm}^{2}$, was notably lower than that utilized in previous plasmonic welding studies [12,17,18,19]. The low input power ensured minimal temperature elevation during the welding process, which is suitable for welding Ag NW films with polymer substrates without significant heat-induced damage. With light irradiation, the sheet resistance of Ag NWs films decreased from 850 to 318 $\Omega sq^{-1}$ after 30 min of exposure, and no further decrease was observed thereafter. Both solvent welding and plasmonic welding methods are gentle approaches for diverse applications, yet their welding efficiencies still need further improvement.

In this research, we proposed a dual combined method of using both solvent and plasmonic welding methods, considering their excellent compatibility in the welding process. By integrating these two strategies, we achieved an enhanced welding efficiency without encountering any adverse effects. In this combined approach, the Ag NW networks were pre-wetted with EG and the projector-based plasmonic welding process was then employed to irradiate the pre-wetted Ag NW networks. Both soaking and irradiation processes were conducted at room temperature. Figure 4A illustrates the change in sheet resistance during the welding process. This novel solvent-based plasmonic welding method substantially reduced the sheet resistance of Ag NW films from 850 to 130 $\Omega sq^{-1}$ over a 15 min period. Notably, the efficiency of this dual approach was better compared to the single solvent welding or plasmonic welding process.

**Figure 5:**
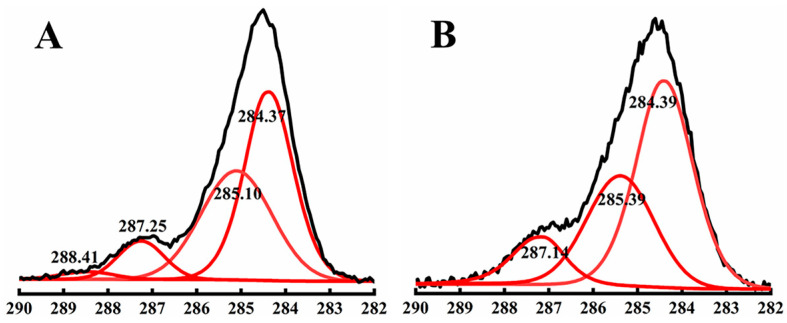
C1s XPS spectra (black lines) and the peak-fitted ones (red lines) of (**A**) Ag NW network before solvent welding, (**B**) Ag NW network after solvent welding.

The SEM images in Figure 4B–D provide a tilted view of Ag NW networks before welding, after 30 min of EG welding, and after solvent-based plasmonic welding, respectively. In Figure 4B, the deposited Ag NWs were piled up on the other Ag NWs before welding. Upon 30 min of EG welding, a slight increase in contact area was observed at the Ag NW junctions (Figure 4C), which might be the reason for the reduction in the sheet resistance of the Ag NW films. Similar changes in Ag NW junctions were observed after 30 min of ethanol welding, depicted in Figure S3. Previous studies have revealed that EG and ethanol can effectively dissolve PVP layers adhering to Ag NWs through van der Waals forces [12]. Decreasing the thickness of residual insulating PVP layers on Ag NW surfaces has been shown to enhance electrical connections at junctions between Ag NWs [9]. XPS measurements were performed to investigate the surface property of Ag NW networks before and after solvent welding process. Before solvent welding, four peaks of 288.41, 287.25, 285.10, and 284.37 eV are fitted as the C 1s envelope, which belong to the agents adsorbed on product surfaces, shown in Figure 5A. The binding energy of 288.41 eV was a characteristic peak corresponding to the carbonyls in CQ, indicating that CQ still absorbs on Ag NW surfaces although it has been cleaned many times with washing and centrifugation in the last step of fabrication for removing residual reactants including surfactants [31]. However, the characteristic peak of CQ disappeared after the solvent welding process, shown in Figure 5B, proving that this solvent welding process can effectively remove residual surfactants. Therefore, the mechanism of solvent welding was proposed to be that EG and ethanol partially dissolved the residual surfactant layers capped on the surface of Ag NWs, leading to increased contact area between Ag NWs at junctions. Additionally, compared with the agglomerated state in air, the free movement of the macromolecular segments of PVP was significantly increased in solvents, thus the PVP had much more freedom to rearrange accordingly to promote the contact between Ag NWs at junctions [37].

Between EG and ethanol, EG was preferred due to its great stability during the welding process and low volatility during the soaking process.

**Figure 6:**
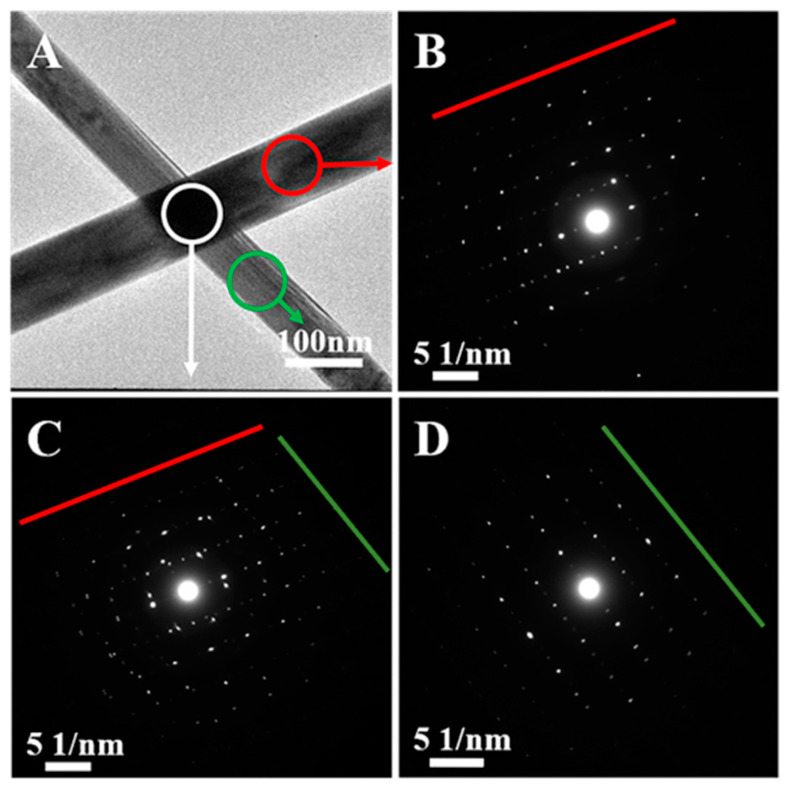
Selected-area electron diffraction (SAED) patterns of a Ag NW network welded using solvent-based plasmonic welding for 30 min. (**A**) Low-magnification TEM image of the Ag NW junction. Circles indicate the approximate location of the diffraction apertures in SAED patterns (**B**–**D**). (**B**) SAED pattern of the upper Ag NWs. (**C**) SAED pattern of the Ag NW junction. (**D**) SAED pattern of the lower Ag NWs.

After 30 min of solvent-based plasmonic welding, the sheet resistance of the Ag NW films was the lowest among the welding methods, in Figure 4A, and the Ag NW contact area at junctions was enlarged, as evident in Figure 4D. Despite the practical welding achieved through the solvent-based plasmonic welding method, no noticeable melting of the Ag NW networks was found. Figure 6 presents the SAED patterns of the Ag NW network junctions welded through solvent-based plasmonic welding for 30 min. In Figure 6B,D, both vital primary diffraction spots and relatively weak double spots, arising from the twinned crystals of Ag NWs, can be found in the top and bottom Ag NWs. The red and green lines mark the parallel lines of spots. At the junction, the double diffraction spots along both directions exhibit roughly equal intensity, indicating no obvious recrystallization was detected after 30 min of solvent-based plasmonic welding, exhibited in Figure 6C. The high-resolution TEM image in Figure S4 also affirmed that the crystal structures of the crossed Ag NWs were not interrupted after solvent-based plasmonic welding, indicating extremely subtle melting of junctions. This phenomenon of slight junction melting after welding was observed in other plasmonic welding processes involving low input power [16,20], indicating that conductivity was significantly enhanced while junction melting was still negligible. The developed welding methods are gentle, effectively avoiding possible damage to the Ag NWs or polymer substrates resulting from overheating. Furthermore, these findings demonstrated that the enhancement in conductivity may arise from removing residual surfactant layers.

**Figure 7:**
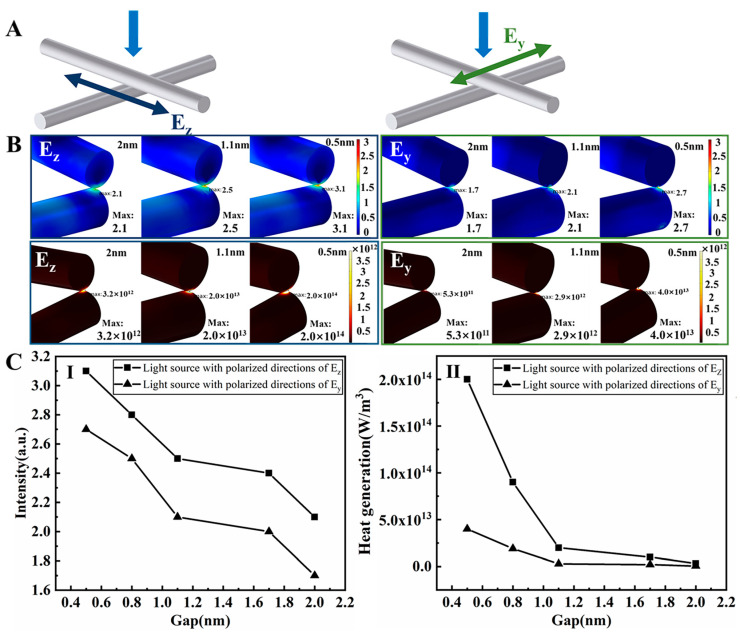
Finite element method simulation depicting the localized surface plasmonic resonance and heat generation of Ag NW junctions illuminated by blue light in the presence of EG. (**A**) Schematic representation of the light polarization in the simulation, either parallel (E_z_) or perpendicular (E_y_) to the top Ag NWs at the junction. (**B**) Illustration of electric field distribution and heat generation at the junction of two orthogonal Ag NWs with gap distances of 0.5, 1.1, and 2 nm. (**C**) Plots showcasing (I) the relationship between maximum electric field intensity and gap distance and (II) the correlation between maximum heat generation and gap distance.

Simulations were employed to investigate the plasmonic welding mechanism, especially the relationship among the electric field, heat generation, and gap distance at the junctions [18,20,38]. However, previous reported simulations only assumed that the Ag NWs were suspended in air, which did not match the conditions during solvent-based plasmonic welding. To further explore the interactions between the solvent and plasmonic welding methods, we conducted simulations of localized surface plasmonic resonance and heat generation at Ag NW junctions using COMSOL Multiphysics 5.0 (Figure S6), simulating both Ag NWs suspended in air and EG. From Figure 7, the maximum electric intensity and heat generation profiles were influenced by both light polarization and the gap distance. Given the random orientations of the Ag NW junctions after spray coating, the light polarization during the welding process was uncontrollable. Consequently, only two representative light polarization directions were investigated.

Figure 7C depicts simulated profiles for the maximum electric intensity and heat generation when the light polarization is parallel (E_z_) or perpendicular (E_y_) to the top Ag NWs in an orthogonally crossed Ag NW junction. Notably, the findings indicated that the E_z_-polarized light was more effective than E_y_-polarized light in achieving welding, with stronger electric fields and increased heat generation resulting from decreased gap distances between Ag NWs, indicating that when Ag NWs were suspended in EG, the trend of electric field and heat generation changed inversely with the gap distance, consistent with situations in which Ag NWs were suspended in air. Previous research often assumed an initial 2 nm gap between Ag NWs, estimated from the surface PVP ligand spacing [19,20]. In our research, the gap distance at junctions could be reduced through the use of EG, which efficiently dissolved residual PVP layers that predominantly determined this distance [12,20]. We proposed that the dissolution of PVP layers by EG leads to a reduction in their thickness, consequently resulting in a decrease in the gap distance. For comparison, we also simulated the cases in which Ag NWs were suspended in air with a fixed 2 nm gap distance to represent the normal plasmonic welding process (Figure S7). When Ag NWs were in air, the E_y_-polarized light was more effective than the E_z_-polarized light, however, the opposite seemed to be valid when Ag NWs were in EG. This behavior was further studied by performing far-field light scattering measurements (Figure S10) which are also discussed in the Supplementary Materials.

When the two wires were separated by a 2 nm gap, estimated based on the 1 nm thickness of the PVP coatings on each Ag NW surface, the maximum normalized electric field intensity was measured at 2.5, and the corresponding maximum heat generation was quantified as $1\times 10^{13}\;W/m^{3}$. These values were higher than those obtained when applying the solvent-based plasmonic welding method with the addition of EG under a 2 nm gap distance (maximum normalized electric field intensity of 2.1, maximum heat generation of $3.2\times 10^{12}\;W/m^{3}$). Although the introduction of EG initially reduced the generated electric field intensity and heat generation at a 2 nm gap distance, the results showed that as the gap distance decreased during the solvent-based plasmonic welding process, both the electric field intensity and heat generation continued to increase. Specifically, when the gap distance decreased to 1.1 nm, the maximum normalized electric field intensity increased to 2.5, while the maximum heat generation was measured at $2.0\times 10^{13}\;W/m^{3}$. Both values were greater than those achieved with a 2 nm gap distance (Figure 7C). Consequently, we proposed that while the addition of EG caused a temporary decrease in the generated electric field intensity and heat generation, this effect was later compensated for by the reduction in gap distance, ultimately making the solvent-based plasmonic welding process an effective welding strategy.

Combining the results from resistance measurements (Figure 4A) with the simulation results (Figure 7), the mechanism of the reduction in resistance during the solvent-based plasmonic welding process can be proposed as follows: (1) blue light induced the plasmonic effect, leading to subtle melting at the Ag NW junctions. (2) The PVP layer on the Ag NWs is dissolved by EG, directly decreasing the resistance among the Ag NWs. (3) The gap distance is reduced owing to the removal of PVP by EG, further enhancing the plasmonic effect. (4) Additionally, the heat generated by the plasmonic effect may contribute to the enhanced dissolution of PVP in the solvent.

Despite the reduction in the sheet resistance of the Ag NW films, the optical transmittance remained nearly unchanged after the dual welding process. This indicated that the welding process did not adversely affect the transmittance of the Ag NW films (Figure S5). To validate the universality of the proposed welding methods, they were also applied to Ag NWs synthesized using only PVP as a surfactant. The corresponding results are presented in Figure S2, demonstrating that the solvent welding and dual welding methods can effectively reduce the sheet resistance of networks fabricated using Ag NWs with thick PVP layers synthesized without the addition of CQ (Figure 3A).

### 3.3. Combined Solvent-Based Plasmonic and Joule Heating Welding of Ag Nanowire Networks

**Figure 8:**
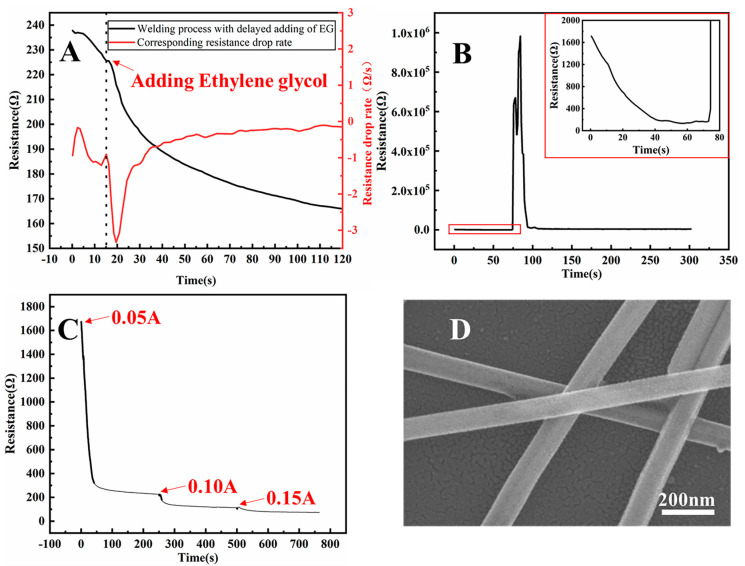
Plots of Ag NW film resistance concerning welding time: (**A**) Real-time changes in resistance caused by adding EG at 15 s and applying a 0.05 A current to the Ag NW film during a blue laser plasmonic welding process (black), along with the corresponding real-time rate of resistance drop (red). (**B**) Real-time change in resistance during an unsuccessful Joule heating welding process with a current of 0.15 A (the inset displays the resistance change in the first 80 s). (**C**) Real-time change in resistance during Joule-heating-enhanced solvent-based plasmonic welding, with gradual increments in current from 0.05 to 0.15 A. (**D**) SEM image of Ag NWs after Joule-heating-enhanced solvent-based plasmonic welding, corresponding to (**C**).

To further investigate the role of EG in reducing the resistance of Ag NW networks during the solvent-based plasmonic welding process, we conducted real-time resistance measurements using a Keithley Digital Multimeter. As depicted in Figure 8A, the rate of resistance reduction notably increased upon the addition of EG, proving the effective enhancement of welding efficiency by EG. The corresponding experimental video for Figure 8A is included in the Supplementary Materials. During the real-time resistance measurements with the Keithley Digital Multimeter, the applied current inevitably induced Joule heating at the junctions of Ag NWs.

While the Joule heating effect has been proposed as an effective strategy to decrease the resistance in Ag NW networks under appropriate conditions, it can lead to overheating and failure of Ag NW networks if not appropriately controlled [22,23]. In this study, upon increasing the current to 0.15 A for Joule heating welding, the resistance of the Ag NW film decreased from 1700 to 200 Ω within 73 s, followed by a sharp increase that indicated the failure of the Ag NW film, as depicted in Figure 8B.

The problem of Joule-heating-induced failure was effectively resolved through our proposed triple welding approach based on combined solvent-based plasmonic and Joule heating welding. In this method, the EG liquid film coated on the Ag NWs acted as an efficient heat dissipation medium, preventing overheating caused by Joule heating. Upon gradually increasing the current from 0.05 to 0.15 A, the combined welding approach reduced the resistance of the Ag NW film from 1676 to 74 Ω within 12.5 min, as shown in Figure 8C. The SEM images of the Ag NW junctions following the triple combined welding in Figure 8C and in Figure 8D demonstrate significantly less melting compared to Ag NW junctions subjected to Joule heating welding with a gradual increase in current from 0.05 to 0.15 A for 12.5 min, as shown in Figure S8. This observation indicated that the presence of EG can effectively prevent the Ag NW junctions from overheating owing to the Joule heating effect. Integrating the Joule heating method into the solvent-based plasmonic welding approach enhanced welding efficiency, and the presence of EG simultaneously improved the welding stability of Ag NWs during Joule heating. In this context, this gentle welding method was applied to weld Ag NW networks deposited on heat-sensitive flexible PET substrates. As shown in Figure S9, the bulb remains illuminated when powered through the flexible Ag NW film, even when the film is bent. This outcome underscores the satisfactory conductivity achieved in Ag NW films after being subjected to this welding method.

## 4. Conclusions

In this study, we developed a CQ-mediated polyol method for synthesizing Ag NWs with thin PVP layers, thus being used to fabricate Ag NW films. Notably, we first developed a solvent welding technique that significantly decreases the resistance of Ag NW films through a straightforward soaking process involving applying an EG or alcohol liquid film on the Ag NW network. The sheet resistance of Ag NW films welded by EG and ethanol decreased from 850 to 268 and 247 $\Omega sq^{-1}$, respectively, after a 15 min treatment. This resistance reduction was attributed to the ability of EG to dissolve residual PVP, thereby increasing the contact area at Ag NW junctions. Based on the solvent welding method, we developed a solvent-based plasmonic welding method that further enhances welding efficiency using low-intensity blue light (60 mW/cm^2^). The sheet resistance of Ag NW films decreased from 850 to 130 $\Omega sq^{-1}$ after 15 min of welding, with the plasmonic effect in promoting Ag NW welding at junctions. The plasmonic effect was proposed to be enhanced by reducing the Ag NW gap distance through removing PVP layers with EG according to the simulation results. To further elevate welding efficiency, we developed a gentle but efficient welding strategy that combines the Joule heating and solvent-based plasmonic welding methods. This approach reduces the resistance of Ag NW networks by approximately 96% to 74 Ω after 12.5 min. In this strategy, EG not only contributes to PVP dissolution but also acts as a heat conductor, dissipating excessive heat generated by Joule heating to prevent overheating. The reported welding method is facile and suitable for industrial fabrication, as the process of soaking, irradiation, and joule heating can be easily assembled. Such a method can effectively weld silver nanowire networks in large areas without using expensive devices or chemical reagents. Also, such a mild welding strategy can prevent silver nanowire and substrate from being damaged during welding, which improves the yield of products. In summary, the developed solvent-based plasmonic and Joule heating welding methods gently and effectively enhance the conductivity of Ag NW networks, making them suitable for applications involving heat-sensitive materials. This research provides a viable strategy for combining different methods to meet stringent requirements in practical applications.

## Data Availability

The data supporting the findings of this study are available by reasonable request to wangxiaolu2019@bjut.edu.cn.

## Supplementary Materials

The following supporting information can be downloaded at: https://www.mdpi.com/article/10.3390/nano13212865/s1, Figure S1: SEM images (A–C) and corresponding statistical diameter distribution (D–F) of Ag NWs synthesized at different molar ratios of CQ to AgNO_3_: (A,D) 1.7, (B,E) 0.9, (C,F) 0.5. All experiments were performed under PVP: AgNO_3_ molar ratio of 1.2:1. Average diameters are indicated in the respective images. Figure S2: The sheet resistance of Ag NW films after different welding times through other welding methods, including plasmonic welding (blue), ethylene glycol welding (red), alcohol welding (green), and solvent-based plasmonic welding (black). The initial sheet resistance of Ag NW films was 850 $\Omega sq^{-1}$. The Ag NW films were fabricated by Ag NWs synthesized with only PVP as capping agent. Figure S3: The SEM images of Ag NW networks welded for 30 min through ethanol welding method. Figure S4: (A) Low-magnification and (B) high-resolution TEM images of Ag NW junction welded by solvent-based plasmonic welding for 30 min. Figure S5: (A) Transmittance of Ag NW films at 550 nm with different initial and final sheet resistances before and after solvent-based plasmonic welding process. (B) Transmittance of Ag NW film with initial sheet resistance of 3000 $\Omega sq^{-1}$ under wavelength of 500–600 nm before (black) and after (red) solvent-based plasmonic welding process. Figure S6: Modeling of silver nanowire for COMSOL simulation. (A) Length and diameter of the Ag NW. (B) Wave direction and the condition around the silver nanowire. Figure S7: Finite element method simulation of the localized surface plasmonic resonance and heat generation of Ag NW junctions illuminated by blue light without ethylene glycol. (A) Schematic of the light polarization during simulation, either parallel (E_z_) or perpendicular (E_y_) to the top Ag NW at junction. (B) Electric field distribution and heat generation at the junction of two orthogonal Ag NWs with gap distance of 2 nm. Figure S8: SEM image of Ag NW junctions after Joule heating welding. Figure S9: Lighting up the bulb through Ag NW film fabricated with PET substrate under (A) unbent state and (B) bent state. Figure S10: Far-field light scattering measurements of a Ag NW junction in air: (A) The light is polarized along the length of the top Ag NW and (B) the light is polarized perpendicularly to the length of top Ag NW. Far-field light scattering measurements of a Ag NW junction in ethylene glycol: (C) The light is polarized along the length of the top Ag NW and (D) the light is polarized perpendicularly to the length of top Ag NW. Video S1: Real-time changes in resistance caused by adding EG at 15 s and applying a 0.05 A current to the Ag NW film during a blue laser plasmonic welding process.
