# Supplementary material for: Solvent Welding-Based Methods Gently and Effectively Enhance the Conductivity of a Silver Nanowire Network
Source: Nanomaterials (Basel). 2023 Oct 29;13(21):2865. doi: 10.3390/nano13212865 (PMC10650926; doi:10.3390/nano13212865)
Supplement: Supplementary file 1 [file nanomaterials-13-02865-s001.zip › nanomaterials-2679020-supplementary.pdf]

Supplementary Materials：

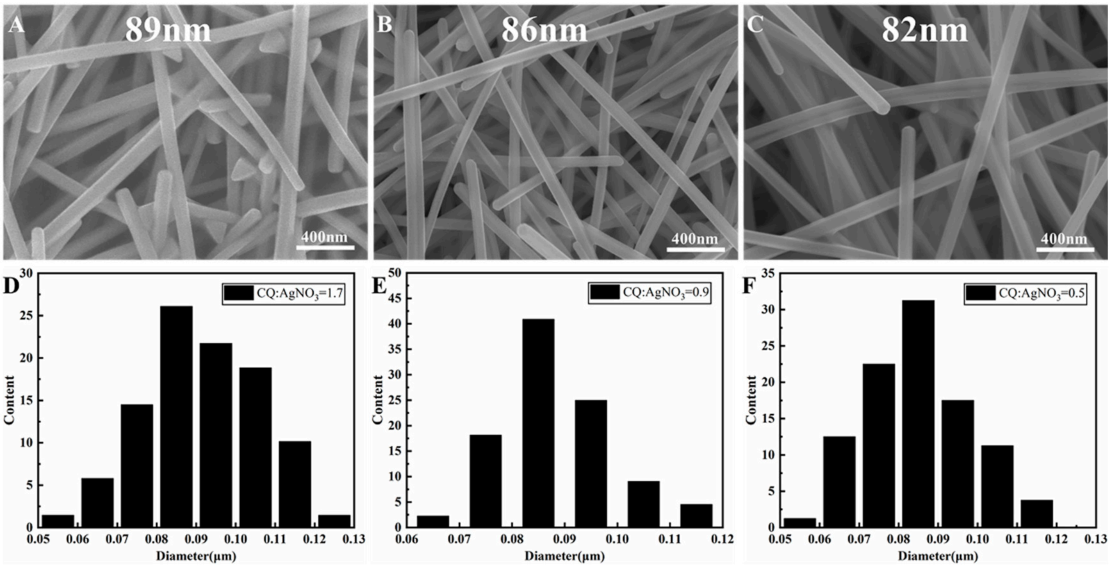

Figure S1.SEM images (A–C) and corresponding statistical diameter distribution (D–F) of Ag NWs synthesized at different molar ratios of CQ to $AgNO_3$: (A, D) 1.7, (B, E) 0.9, (C, F) 0.5. All experiments were performed under PVP: $AgNO_3$ molar ratio of 1.2:1. Average diameters are indicated on the respective images.

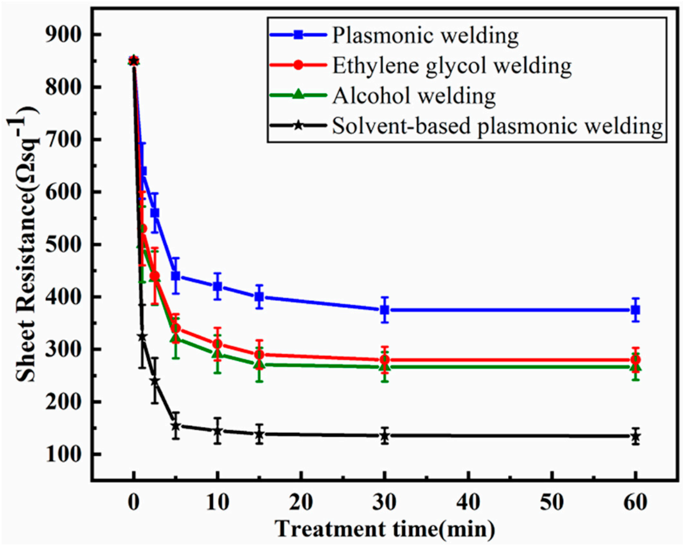

Figure S2. The sheet resistance of Ag NW films after different welding time through other welding methods, including plasmonic welding (blue), ethylene glycol welding (red), alcohol welding (green) and solvent-based plasmonic welding (black). The initial sheet resistance of Ag NW films was 850 Ωsq^{-1}. The Ag NW films were fabricated by Ag NWs synthesized with only PVP as capping agent.

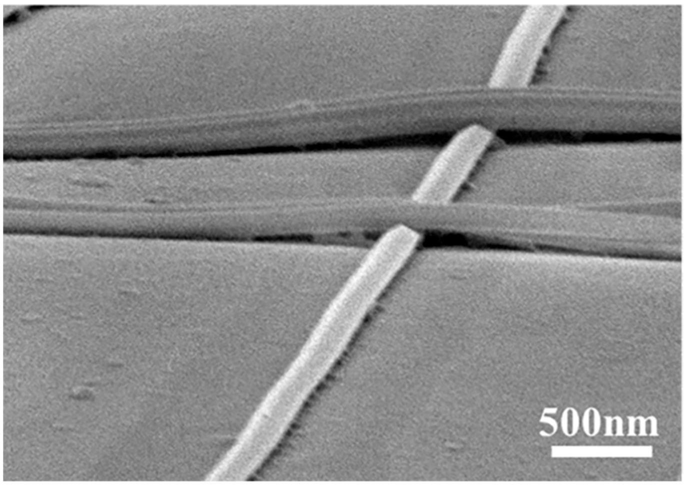

Figure S3. The SEM images of Ag NW networks welded for 30min through ethanol welding method.

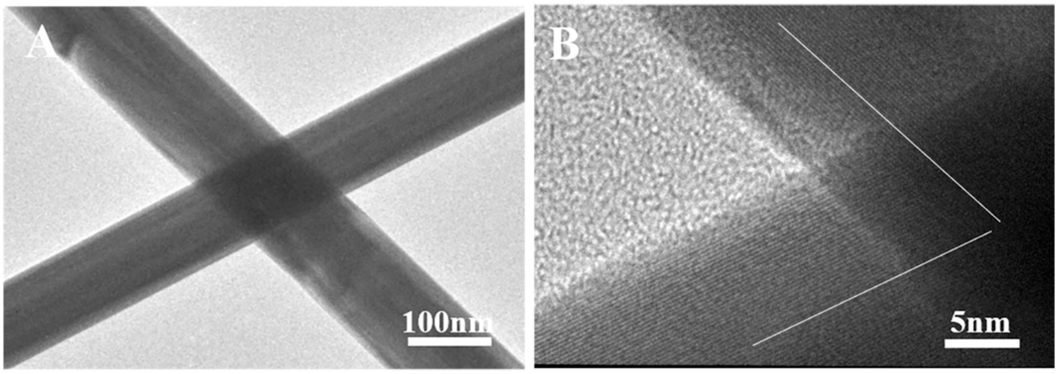

Figure S4. (A) Low-magnification and (B) high-resolution TEM images of Ag NW junction welded by solvent-based plasmonic welding for 30min.

Given that the uniformity of Ag NW networks deposited through the spray-coating remains an area for improvement in this study, films with original sheet resistances ranging from 1470 to 4600 Ωsq^{-1} were found to exhibit similar deposition quantities of Ag NWs. This similarity was deduced from their comparable transmittances at 550 nm, ranging between 80.2 and 83.9, as shown in Figure S5(A). It was proposed that the reason why sheet resistances of films with similar deposition quantities shows large differences was because of the unevenly distributed of Ag NW networks. Employing the combined welding method, Ag NWs films with varying original sheet resistances were processed to achieve final sheet resistances between 251 and 353 Ωsq^{-1}. This outcome indicates that the combined welding approach remains robust even when processing unevenly distributed Ag NW networks. Notably, the transmittance of Ag NW films experienced only minimal alteration after solvent-based plasmonic welding, as depicted in Figure S5(A). As representative data, the transmittance of a Ag NW film with an initial sheet resistance of 3000 Ωsq^{-1} measured across different wavelengths is displayed in Figure S5(B). This visualization underscores that the solvent-based plasmonic welding method has negligible impact on the transmittance of Ag NW films.

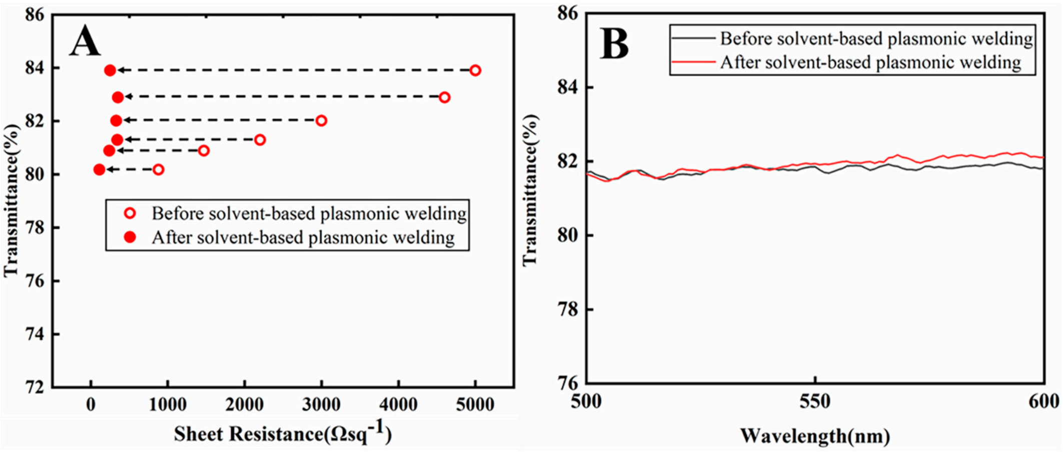

Figure S5. (A) Transmittance of Ag NW films at 550nm with different initial and final sheet resistance before and after solvent-based plasmonic welding process. (B) Transmittance of Ag NW film with initial sheet resistance of $3000\Omega sq^{-1}$ under wavelength of 500-600nm before (black) and after (red) solvent-based plasmonic welding process.

To elucidate the underlying mechanism of the welding process, we employed the commercial COMSOL program for simulation purposes. In this simulation, two Ag NWs were modeled with diameters of 82 nm and lengths of 1100 nm. These Ag NWs were positioned orthogonally to each other, separated by a distance of 0.5–2 nm, reflecting the ligand spacing of the polymer binder around the NWs (Figure S6(A)). As depicted in Figure S6(B), vertically intersecting nanowires were suspended either in air or an EG layer to respectively simulate the welding processes without or with EG. The incident light was propagated as a plain wave along the positive x-direction, perpendicular to the plane of the Ag NWs. Furthermore, this incident light was characterized by z- and y-polarized electric fields, with a wavelength of 445 nm and an intensity of 60 mW/cm^2. The interaction between light and nanostructure is described by the classical Maxwell's equations, and the electric field distribution can be calculated from the Helmholtz wave equation (1), as derived from the Maxwell's equations[32].

$$\nabla \times \mu_r^{-1}(\nabla \times E) - \left(\frac{\omega}{c_0}\right)^2 \varepsilon_m E = 0 \quad (1)$$

We suppose that $|E| \propto exp(-i\omega t)$, where ω is the angular frequency of incoming light, μ_r is the magnetic permeability, ε_m is the permittivity of the surrounding medium.

The generated heat induced by light absorption could be simulated by the equation (2) and was directly related to the imaginary part of the dielectric constant of material[33,34].

$$Q_d = \frac{1}{2}\varepsilon_0 \omega\, Im(\varepsilon_r)|E|^2 \quad (2)$$

ε_0 is permittivity of vacuum; ω is angular frequency of the light; ε_r is the relative permittivity of silver; E is electric field.

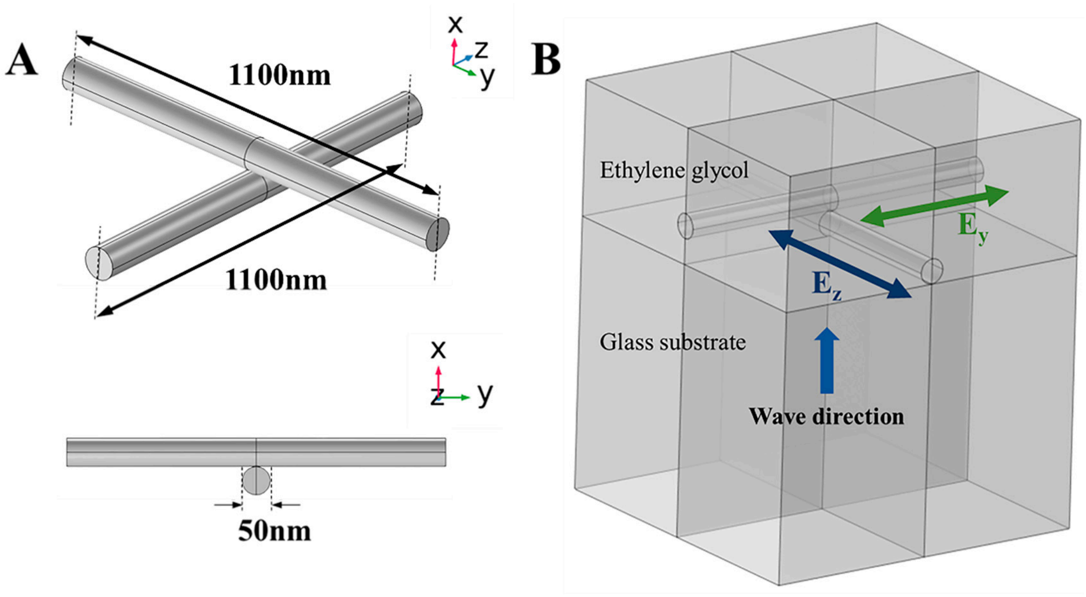

Figure S6. Modeling of silver nanowire for COMSOL simulation. (A) Length and diameter of the Ag NW. (B) Wave direction and the condition around the silver nanowire.

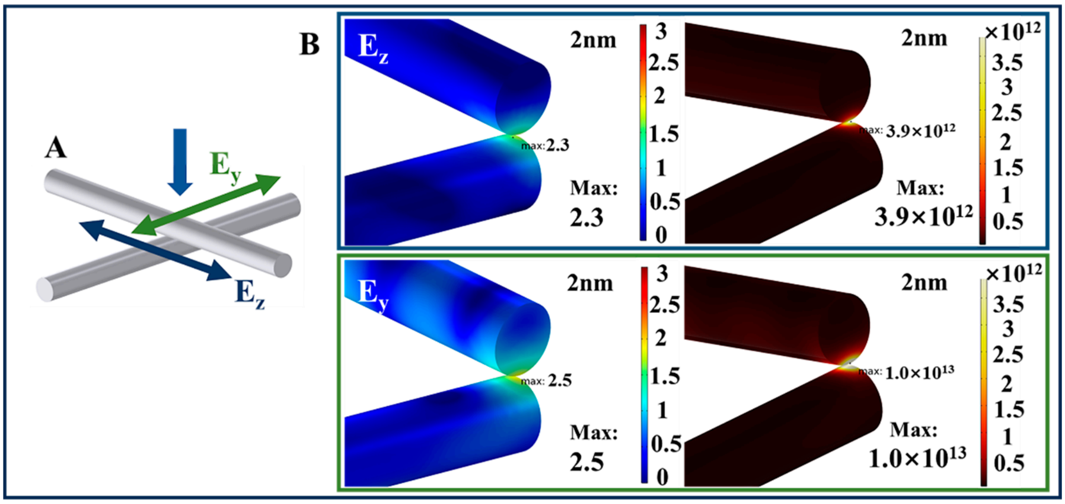

Figure S7. Finite element method simulation of the localized surface plasmonic resonance and heat generation of Ag NW junctions illuminated by blue light without ethylene glycol. (A)Schematic of the light polarization during simulation, ether parallel (Ez) or perpendicular (Ey) to the top Ag NW at junction. (B) Electric field distribution and heat generation at the junction of two orthogonal Ag NWs with gap distance of 2nm.

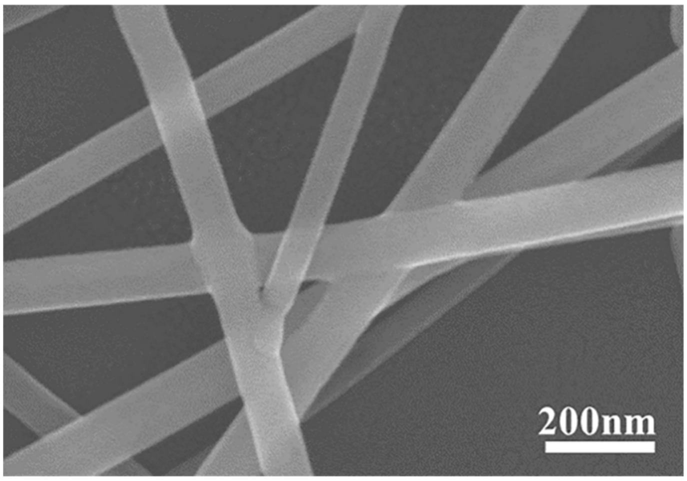

Figure S8. SEM image of Ag NW junctions after Joule heating welding.

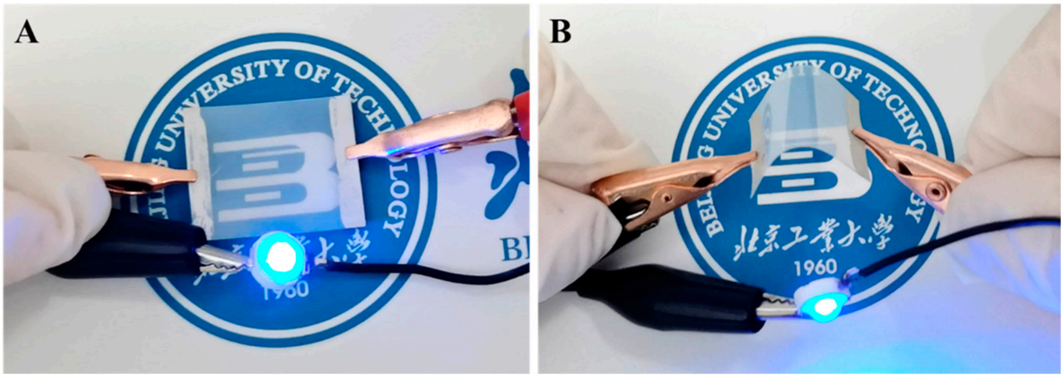

Figure S9. Lighting up the bulb through Ag NW film fabricated with PET substrate under (A)unbent state and (B)bent state.

The simulation results in our research show that, when the surrounding material of Ag NWs is air, the E_y-polarized light is more effective than the E_z-polarized light. This trend is consistent with that reported in other researches[16,20]. The mechanism of this phenomenon has proposed to be that when the light is polarized along the top nanowire, a localized surface plasmon resonance cannot be excited in this wire and instead the light is strongly backscattered, reducing field penetration into the junction and leading to less heat generation. The observations are consistent with the far-field light scattering measurements, in which a clear difference in scattering contrast was found when light was polarized along or perpendicular to a nanowire[16]. We have made the same far-field light scattering measurements, as shown in Figure S10. When in air, the observations shown in Figure S10 (A,B) are consistent with that in the literature[16,20]. However, when in ethylene glycol, as can be seen from Figure S10 (C,D), that upon tunning the polarization direction of light the trend of scattering contrast is opposite to that in air. For example, comparing Figure S10 (A) to Figure S10 (C), in which the polarizing directions are the same, the Ag NW which is bright turns from the top one to the bottom one. This well indicates that ethylene glycol and air affect the polarization direction of light in an opposite way. This is consistent with the simulation result (Figure 7). We propose that possible reason of this behavior may attribute to much higher reflective index and dielectric constant of

ethylene glycol than that of air.

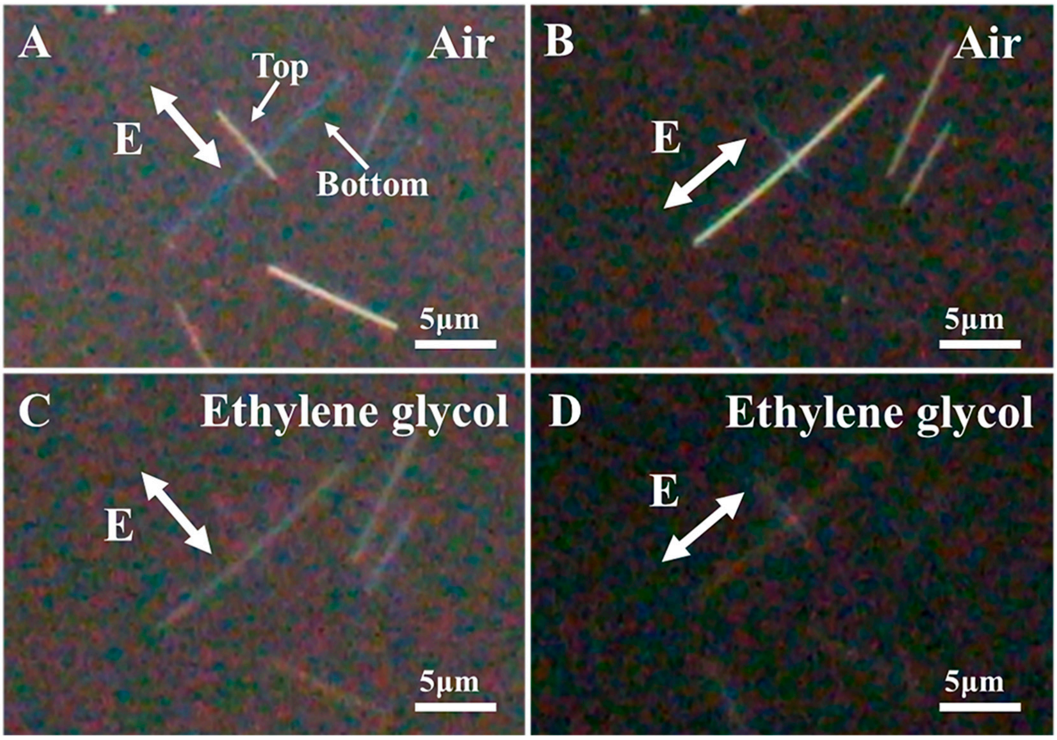

Figure S10. Far-field light scattering measurements of a Ag NW junction in air: (A) the light is polarized along the length of the top Ag NW and (B) the light is polarized perpendicularly to the length of top Ag NW. Far-field light scattering measurements of a Ag NW junction in ethylene glycol: (C) the light is polarized along the length of the top Ag NW and (D) the light is polarized perpendicularly to the length of top Ag NW.
