# Supplementary figures and images for: Solvent Welding-Based Methods Gently and Effectively Enhance the Conductivity of a Silver Nanowire Network
Source: Nanomaterials (Basel). 2023 Oct 29;13(21):2865. doi: 10.3390/nano13212865 (PMC10650926; doi:10.3390/nano13212865)

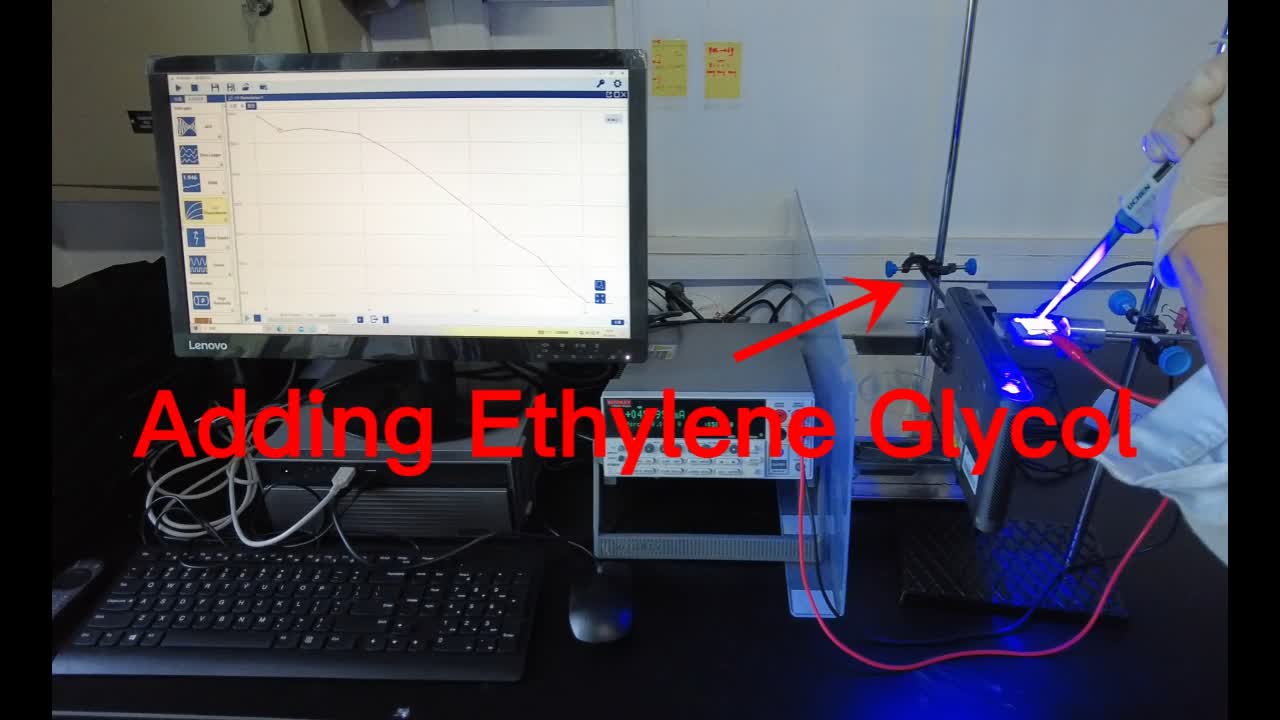

Supplement: Supplementary file 1 [file nanomaterials-13-02865-s001.zip › video still.jpg]
